# Dr. James G. A. Davies

**Published:** 1917-12

**Authors:** 


### OBITUARY

**Readers are requested to report promptly the death of all physicians in Western New York, or former residents of this region, or graduates of any medical school in Western New York, and to notify the families of the deceased of our desire to publish adequate Obituary notices.**

---

**Dr. James G. A. Davies,** Druidic of Me., 1882, of Dalton, N. Y., is reported dead by the P. O. We have seen no notice of his death since his name was listed in the 1916 edition of the State Directory and have been unable to elicit information by correspondence. Will any of our readers confirm the report or furnish details?

